# Distinct transcriptomic signatures define febrile malaria depending on initial infective states, asymptomatic or uninfected

**DOI:** 10.1186/s12879-024-08973-2

**Published:** 2024-01-29

**Authors:** Kelvin M. Kimenyi, Mercy Y. Akinyi, Kioko Mwikali, Tegan Gilmore, Shaban Mwangi, Elisha Omer, Bonface Gichuki, Juliana Wambua, James Njunge, George Obiero, Philip Bejon, Jean Langhorne, Abdirahman Abdi, Lynette Isabella Ochola-Oyier

**Affiliations:** 1 KEMRI‑Wellcome Trust Research Programme, Kilifi, Kenya; 2 Department of Biochemistry, University of Nairobi, Nairobi, Kenya; 3 Centre for Tropical Medicine and Global Health, Nuffield Department of Medicine, University of Oxford, Oxford, UK; 4 The Francis Crick Institute, London, UK

**Keywords:** Malaria, Asymptomatic *Plasmodium falciparum* infection, Immunity, Host-parasite interactions

## Abstract

**Background:**

Cumulative malaria parasite exposure in endemic regions often results in the acquisition of partial immunity and asymptomatic infections. There is limited information on how host-parasite interactions mediate the maintenance of chronic symptomless infections that sustain malaria transmission.

**Methods:**

Here, we determined the gene expression profiles of the parasite population and the corresponding host peripheral blood mononuclear cells (PBMCs) from 21 children (< 15 years). We compared children who were defined as uninfected, asymptomatic and those with febrile malaria.

**Results:**

Children with asymptomatic infections had a parasite transcriptional profile characterized by a bias toward trophozoite stage (~ 12 h-post invasion) parasites and low parasite levels, while early ring stage parasites were characteristic of febrile malaria. The host response of asymptomatic children was characterized by downregulated transcription of genes associated with inflammatory responses, compared with children with febrile malaria,. Interestingly, the host responses during febrile infections that followed an asymptomatic infection featured stronger inflammatory responses, whereas the febrile host responses from previously uninfected children featured increased humoral immune responses.

**Conclusions:**

The priming effect of prior asymptomatic infection may explain the blunted acquisition of antibody responses seen to malaria antigens following natural exposure or vaccination in malaria endemic areas.

**Supplementary Information:**

The online version contains supplementary material available at 10.1186/s12879-024-08973-2.

## Introduction

Malaria remains a global health concern that was responsible for 247 million cases and 619,000 deaths in 2021 [1], despite the availability of a working diagnostic test, effective treatment and the recently licenced vaccine. *Plasmodium falciparum* is the main causative agent of malaria infections in sub-Saharan Africa. During the parasite’s life cycle, the merozoite stage that emerges from the liver invades new erythrocytes, developing into rings, trophozoites and finally into multinucleated schizonts containing new merozoites that are released to perpetuate the life cycle [2]. The bursting of infected erythrocytes releases merozoites and parasite byproducts that cause fever, a characteristic feature of clinical malaria [3]. In addition, later stages of parasite development (trophozoites and schizonts) in infected erythrocytes introduce parasite proteins, termed variant surface antigens (VSAs), on the erythrocyte cell membrane facilitating cytoadherence. Cytoadherence is the binding, through VSAs, of the infected erythrocyte onto host endothelial cells, which influences parasite virulence and malaria disease severity [4]. Therefore, in the case of asymptomatic infections reduced parasite cytoadherence and increased circulation of parasites in peripheral blood is suggested to be the mechanism maintaining asymptomatic infections, due to the reduction in parasitemia to a level that is tolerable to the immune system [5]. The complex range of host-parasite interactions results in a spectrum of clinical presentations of *P. falciparum* infections from severe, through uncomplicated febrile episodes, to asymptomatic parasite carriers [6, 7].

Due to repeated exposure to malaria, individuals residing in endemic regions acquire partial immunity or are semi-immune to malaria, enabling them to control parasitemia and inflammation [8, 9]. However, this natural partial immunity rarely progresses to sterile immunity and thus individuals are continually infected with the parasite, often resulting in asymptomatic infections [10]. The immunity in asymptomatic individuals has been summarized as antiparasitic immunity that prevents parasite growth and anti-disease immunity that prevents symptoms [11]. However, this advantage of partial immunity is lost in individuals who leave malaria endemic regions [12]. Asymptomatic infections are problematic for malaria control efforts as they often go undetected and act as a reservoir of parasites that perpetuate malaria transmission [6]. They have also been shown to increase the risk of febrile illness in low transmission settings and appear to be protective against febrile disease in high transmission settings [13–15]. Additionally, they may be precursors of symptomatic malaria primarily due to superinfections with new parasite clones [16–19]. Therefore, immune tolerance to the parasite clones circulating during an asymptomatic infection potentially reduces the risk of subsequent febrile infections with the same clone [20]. Furthermore, prior malaria exposure may interfere with vaccine efficacy as malaria exposed adults have been shown to elicit reduced immune responses, particularly antibodies, compared to infants and malaria naïve adults [21, 22].

Asymptomatic malaria infections have previously been explored using transcriptomic studies, which revealed reduced transcription of pro-inflammatory genes [23], inhibition of T-cell function [24] and enhanced p53 pathway expression by monocytes [25]. Other studies have associated these infections with greater levels of immunoregulatory cytokines [26, 27]. Despite the rapid increase in knowledge obtained from clinical malaria isolates, much less is known about gene expression by the parasite and host immune cells during asymptomatic *P. falciparum* infections and the ensuing follow-up febrile infections. In this study, we followed a longitudinal cohort of children with similar history of exposure to infection and sought to understand the biology of asymptomatic infections. We tracked within-host responses and defined, at a single snapshot in time, asymptomatic and uninfected individuals and their ensuing febrile infection pairs. A comparison of the parasite transcriptome and the host PBMCs transcriptome and proteome was conducted between the paired asymptomatic-febrile and uninfected-febrile episodes. The population differences between uninfected, asymptomatic and febrile individuals were also examined. Thus, this study was set out to determine whether detectable asymptomatic infections had an impact on the stages of the parasite in circulation, whether the host immune response differed when compared to that of children from the same cohort with no detectable parasitaemia (uninfected), and whether asymptomatic infection affects the host response to a febrile superinfection.

## Methods

### Study population

This study was conducted in Junju, a moderate to high malaria transmission region in Kilifi County, Kenya. This region experiences two rainy seasons annually, the long rains in April to July and the short rains in October to November, which are characterised by increased malaria transmission [28]. The samples were collected from a cohort of 425 children living in Junju who were recruited at birth and followed up weekly by active surveillance until the age of 15, where *P. falciparum* malaria was diagnosed (using a rapid diagnostic test (RDT) and confirmed with microscopy) and treated [29]. Annual cross-sectional blood surveys were conducted in this cohort before the long rains from 2007 to 2018 and the children were defined as uninfected, asymptomatic, febrile malaria and non-malarial fever.

### Definitions

**Fig. 1:**
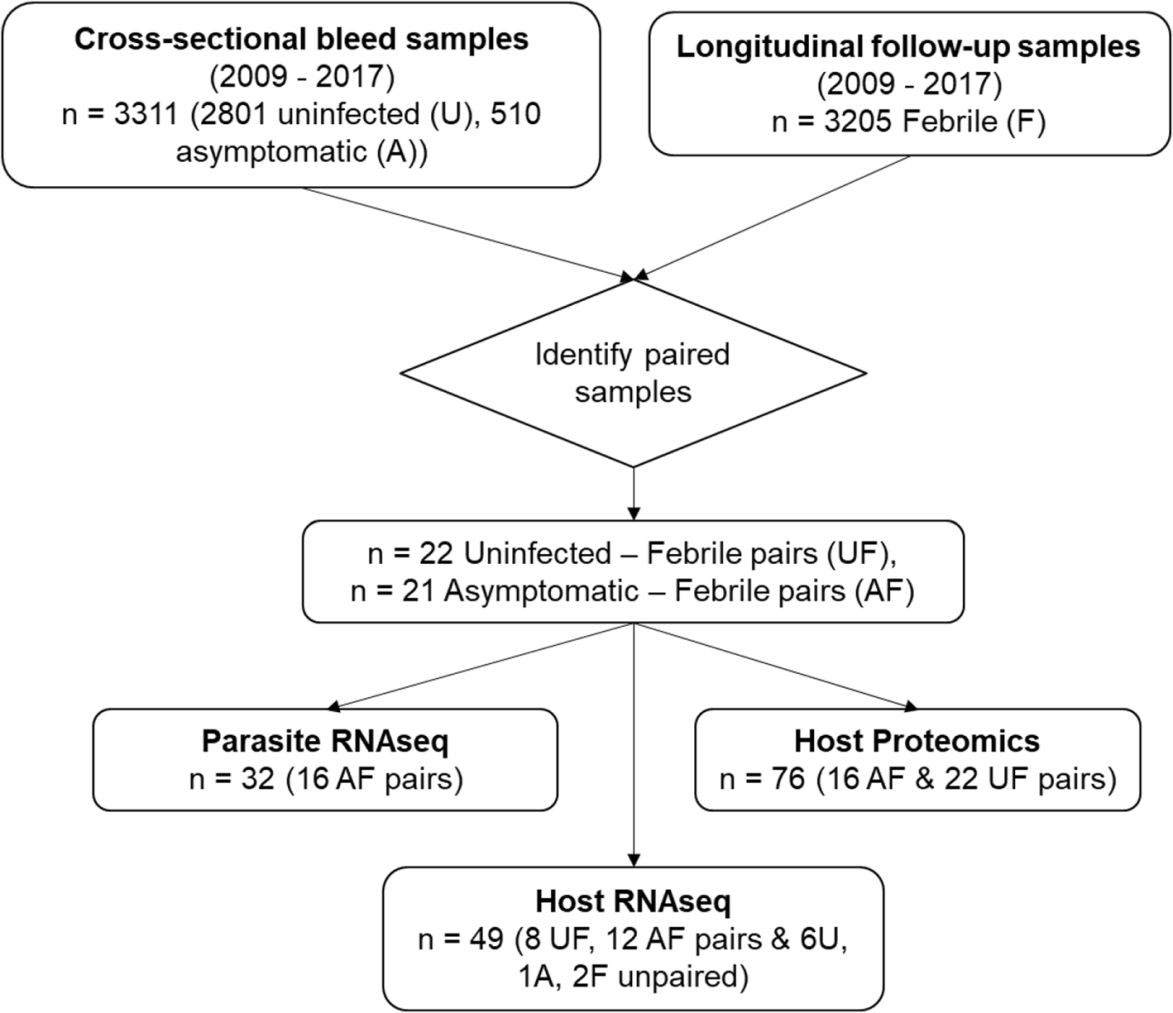
A schematic of the sample selection process. Uninfected (U) (*P. falciparum* negative and no malaria symptoms) and asymptomatic (A) (*P. falciparum* positive and no malaria symptoms) samples were collected during the annual cross-sectional bleed surveys between 2007 and 2017. Subsequent febrile malaria episodes (F) (*P. falciparum* parasitemia ≥ 2500 parasites/µl and axillary temperature ≥ 37.5 °C) were detected during weekly active surveillance (longitudinal follow-up). *P. falciparum* detection was based on microscopy. Whole blood from all the sample groups were processed and peripheral blood mononuclear cells (PBMCs) isolated. *P. falciparum* infected red blood cells (RBCs) were processed from the A and AF paired samples

Uninfected children were defined as being parasite negative by RDT and microscopy. Asymptomatic infections were parasite positive by RDT, and confirmed by microscopy, with an axillary temperature < 37.5 °C and no symptoms of fever, and with no febrile malaria episode within the month up to the survey or the subsequent 7 days after the date of the survey [13, 16]. Sub-microscopic asymptomatic infections were excluded as they yielded insufficient RNA material for RNAseq during optimization assays. For the first-febrile malaria infections from individuals who were initially uninfected their parasitemia was defined as ≥ 2500 parasites/µl by microscopy and a tympanic temperature ≥ 37.5 °C based on definitions previously described for this cohort [27] while any parasitaemia by microscopy and a tympanic temperature ≥ 37.5 °C was considered for the first-febrile infections from those who were initially asymptomatic. Only microscopy-positive samples were included in this study. A total of 2801 uninfected, 510 asymptomatic and 3205 febrile samples collected between 2009 and 2017 were selected for subsequent analysis. About 154 paired samples from children who were asymptomatically infected on the survey and subsequently experienced febrile infections were identified for parasite and PBMC transcriptomic and proteomic analysis but only 21 pairs were available in the biobank. Additionally, 22 paired uninfected and corresponding febrile infections were available for PBMC transcriptomic and proteomic analysis out of 1500 pairs identified in the database (Fig. 1).

### Sample collection

Blood samples were drawn by venipuncture from all the participants during the cross-sectional bleed and at their follow-up febrile malaria episode. The samples were collected in sodium citrate-containing cell preparation tubes (Vacutainer CPT Tubes, BD) and transported to the laboratory where they were separated into peripheral blood mononuclear cells (PBMCs) and red blood cell (RBC) pellets, harvested and processed before storage in liquid nitrogen (LN_2_). PBMCs were isolated using the Lymphoprep™ density gradient solution, carefully collected using a wide mouth Pasteur pipette and then washed twice with RPMI media before storage in LN_2_. Each sample was then separated into two components, one for transcriptomics and the other for proteomics.

### Preparation and sequencing of *P. falciparum* RNA

At the time of the study, infected erythrocytes from the 21 asymptomatic-febrile sample pairs were retrieved and thawed using decreasing concentrations of NaCl (16% (w/v), 1.2% (w/v) and 0.9% (w/v)) to remove the glycerolyte storage medium. The samples were digested using 0.02% (w/v) saponin, and the parasites were separated from RBC components by centrifugation. RNA was extracted using the ISOLATE II RNA Mini Kit (Bioline) and eluted into 40 µl RNAse-free water. The RNA quality, concentration and purity were assessed using NanoDrop 2000c (Thermo Scientific). mRNA was enriched using the NEBNext™ Poly(A) mRNA magnetic isolation module, and first strand cDNA was synthesized using Superscript III reverse transcriptase (Invitrogen), while the second strand was synthesized using the NEBNext™ RNA Second Strand Synthesis Module (New England Biolabs). Next, 13.5 µl of cDNA was prepared into libraries using the NEBNext™ Ultra II FS DNA library preparation kit (New England Biolabs) and amplified in 17 cycles using the KAPA Library Amp Primer mix (KAPA Biosystems) to increase yield. The Agilent DNA 1000 chips on the Agilent TapeStation 2200 system were used to assess the quality and concentration of the libraries, which were then quantified using the KAPA Library Quantification Kit – Complete Kit (ABI Prism) and pooled into equimolar amounts. Paired-end sequencing was performed using the Illumina HiSeq 4000 platform at the Wellcome Sanger Institute (WSI), UK.

Raw sequence reads in fastq file format were assessed for quality and mapped to the reference *P. falciparum* (3D7 strain, PlasmoDB, release 55) genome using Kallisto’s v0.46.1 [28] default settings. Only genes with more than 2 counts per million in 10 or more samples were retained. Parasite age hours post-invasion (hpi) was determined using the maximum likelihood estimation method described in [29] with reference data from [30]. Proportions of parasite stages per sample were determined using a mixture model adapted from [31] using reference data from López-Barragán et al., (2011) [32]. Differences between stage proportions of paired samples were analysed using the Wilcoxon rank sum test. Pearson correlation was used to associate the proportion of rings and nonrings with parasitemia. Normalization was performed using the trimmed mean of M values (TMM) normalization in edgeR v3.38.1 [33] and unwanted variation factors were estimated using RUVSeq v1.30.0 [34].

### Transcriptional analysis of host PBMCs

RNA was extracted from host PBMCs from 21 asymptomatic-febrile pairs and 22 uninfected-febrile pairs of host PBMCs and libraries prepared using the same protocol as the parasite samples described above. Paired-end sequencing was performed on a NovaSeq 6000 (Illumina) system at the Advanced Sequencing Facility at The Francis Crick Institute, UK. The raw fastq files generated were processed using the RNA-Seq pipeline from the NF-core framework (v21.10.6) [35] using STAR (v2.7.10a) [36] aligner and RSEM (v1.3.1) [37] quantification and all other parameters as default. Samples were mapped to the human reference genome version GRCh38 release 95 obtained from Ensembl. Only samples with greater than 10 million aligned reads were considered. Hemoglobin genes described in [38] were removed from the analysis. Genes having 5 counts per million or higher in 13 (number of samples in the least popular group) or more samples were retained and normalized using TMM normalization. To determine differentially expressed genes among the uninfected, asymptomatic and febrile sample groups, gene analysis was performed by fitting a negative binomial generalized log-linear model in edgeR v3.38.1 [33]. The following matrix formula was applied to the edgeR model: ~ 0 + treatment + pair + batch. Here, ‘treatment’ aligned to either uninfected, asymptomatic or febrile, ‘pair’ indicated the pairing of samples per individual; and ‘batch’ represented the RNA processing batch. Differentially expressed genes were partitioned into four clusters with the least intracluster variation using K-means clustering and plotted using a heatmap. Additional pairwise analyses were conducted to identify differences between two sample groups. Genes in each cluster were used as input during functional overrepresentation analysis to identify enriched pathways using Gene Ontology (GO) and Kyoto Encyclopedia of Genes and Genomes (KEGG) gene sets [39] in clusterProfiler v4.4.4 [40]. Pathways below a Benjamin-Hochberg adjusted *P* value cut of 0.05 were retained. Deconvolution of log-transformed and normalized counts was performed in CIBERSORT v1.04 [41] using the LM22 signature gene set as a reference to determine the proportion of each immune cell per sample [42]. The differences in cellular proportions among the four conditions were then examined.

### Proteomics analysis of PBMCs

Proteins were extracted from PBMCs by resuspending the pellet with 5 µl of 6 M UREA (Thermo Scientific). The protein samples were then adjusted with 50 mM triethylamonium bicarbonate (TEAB, Sigma-Aldrich) to 100 µl and the protein concentration was determined using the bicinchoninic acid (BCA) protein assay (Thermo Scientific). The protein samples were then reduced with 40 mM dithiothretol, alkylated with 80 mM iodoacetamide in the dark, and quenched with 80 mM iodoacetamide at room temperature, followed by digestion with1 µg/µl of trypsin [43]. Nine pools, each containing 9 samples and 1 control for batch correction, were prepared by combining 1 µl aliquots from each sample. The samples were pooled using a custom randomization R script. The pooled samples were then individually labelled using the Tandem Mass Tag (TMT) 10-plex kit (Thermo Scientific) according to the manufacturer’s instructions. One isobaric tag was used solely for the pooled samples and combined with peptide samples labelled with the remaining 9 tags. The labelled peptide pools were then desalted using P10 C18 pipette ZipTips (Millipore) according to the manufacturer’s instructions. Eluted peptides were dried in a Speedvac concentrator (Thermo Scientific) and resuspended in 15 μl loading solvent (98% H_2_O, 2% acetonitrile, 0.05% formic acid). The peptides were then quantified using a Qubit Protein Assay Kit (Thermo Fisher Scientific) according to the manufacturer’s instructions. A standardized protein concentration of 5 µg was finally injected into the LC–MS/MS for analysis. The peptides were then loaded onto the liquid chromatograph, separated on reverse-phase analytical column of 75 µm x 50 cm C18 (Thermo Scientific) and measured using a Q Exactive Orbitrap mass spectrometer as described by [43]. To identify and quantify proteins, mass spectrometer output files were analyzed using MaxQuant software version 2.0.3.0 [44] by searching against the UniProt human proteome (downloaded on 10/06/2021) using the Andromeda search engine [45]. N-terminal acetylation and methionine oxidations were set as variable modifications while cysteine carbamidomethylation and TMT-10plex labelled N-terminus and lysine were set as fixed modifications. The false discovery rate (FDR) cut-off was set as 0.01 for both proteins and peptides with a minimum length of seven amino acids and was determined by searching a reverse database. Enzyme specificity was set as C-terminal to arginine and lysine with trypsin as the protease. Only up to two missed cleavages were allowed in the database search. Peptide identification was performed with an allowed fragment mass deviation maximum of 20 ppm (parts per million) and an initial precursor mass deviation maximum of 7 ppm. Default parameters for Orbitrap-type data were used. The pooled sample channels were used for batch correction. The 10-plex corrected reported ion intensity matrix was extracted from the protein group output file and used for downstream analysis. Proteins matching the reversed part of a decoy database, potential contaminants and proteins only identified by a modification site were excluded. Differential protein abundance analysis of the labelled sample intensities generated by MaxQuant was performed using PERSEUS v2.05.0 MaxQuant software (MaxPlanck Institute of Biochemistry, Martinsried, Germany) as described in [46]. The differentially expressed proteins were loaded in STRING version 11.5 (https://string-db.org/) databases for protein–protein interaction and Gene Ontology (GO) functional analyses. Statistical significance was set at FDR < 0.05.

## Results

### Malaria case burden in the cohort

**Fig. 2:**
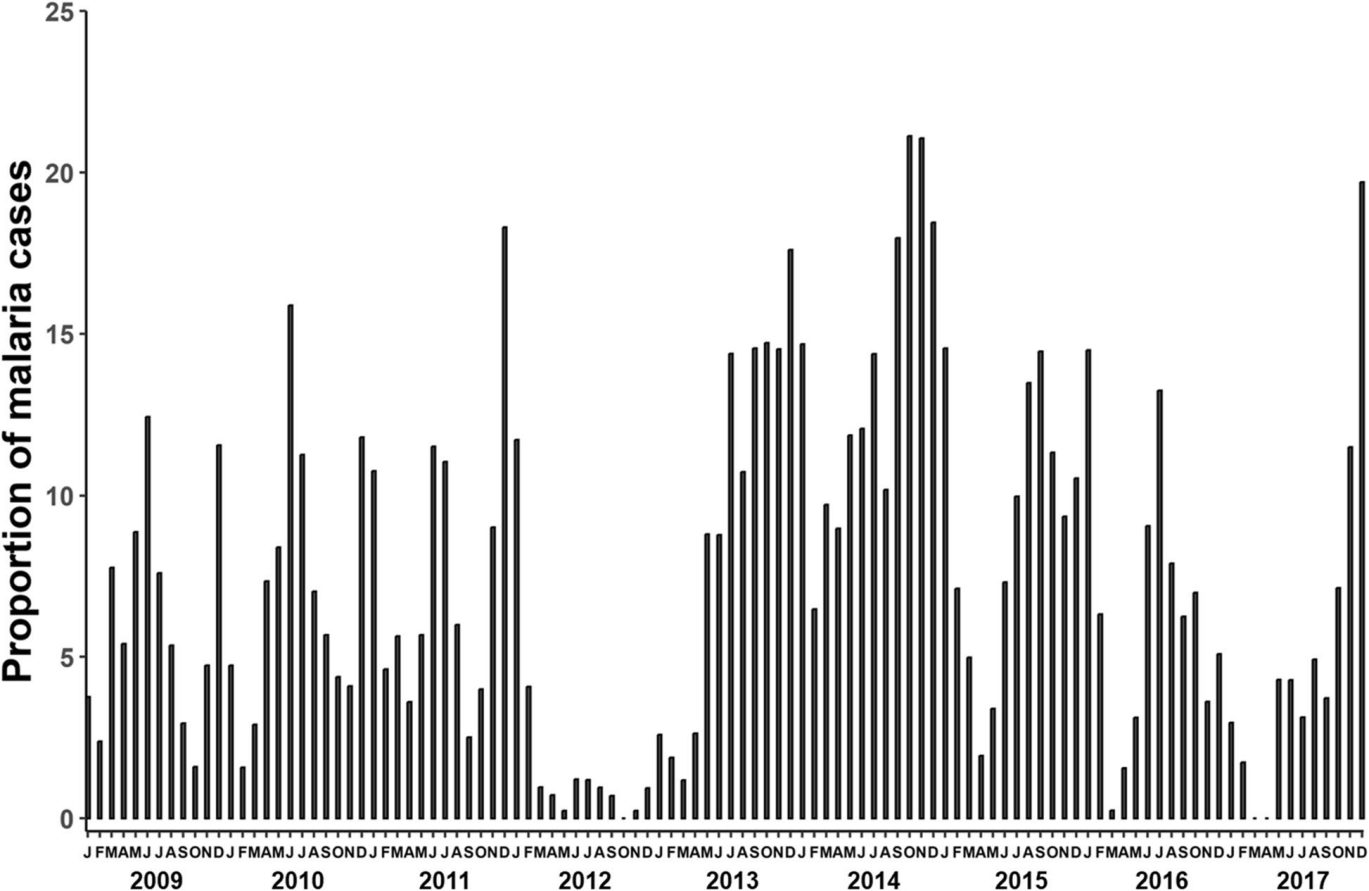
Perennial malaria transmission in the Junju cohort. The proportion of symptomatic malaria cases (diagnosed by presence of fever (temperature ≥ 37.5^0^C) and parasitemia ≥ 2500 parasites/µl) recorded in the cohort during the weekly active surveillance. The proportion of malaria cases is the percentage of malaria cases reported per month. The samples were collected from ~ 425 children aged between 0 and 15 years recruited in the Junju cohort

Between 2009 and 2017, the cohort’s distribution of febrile malaria cases throughout the year showed perennial malaria transmission, with at least 2 peak months per year. The overall trend in malaria cases was consistent, but there was a drastic drop in the prevalence in malaria cases in 2012 (Fig. 2). The proportion of *P. falciparum* positive asymptomatic infections during the cross-sectional bleed from 2009 to 2017 was highest in 2010 at 25.2% and lowest in 2017 at 4.2%.

### Differential parasite expression profiles of asymptomatic infections compared with febrile malaria

**Fig. 3:**
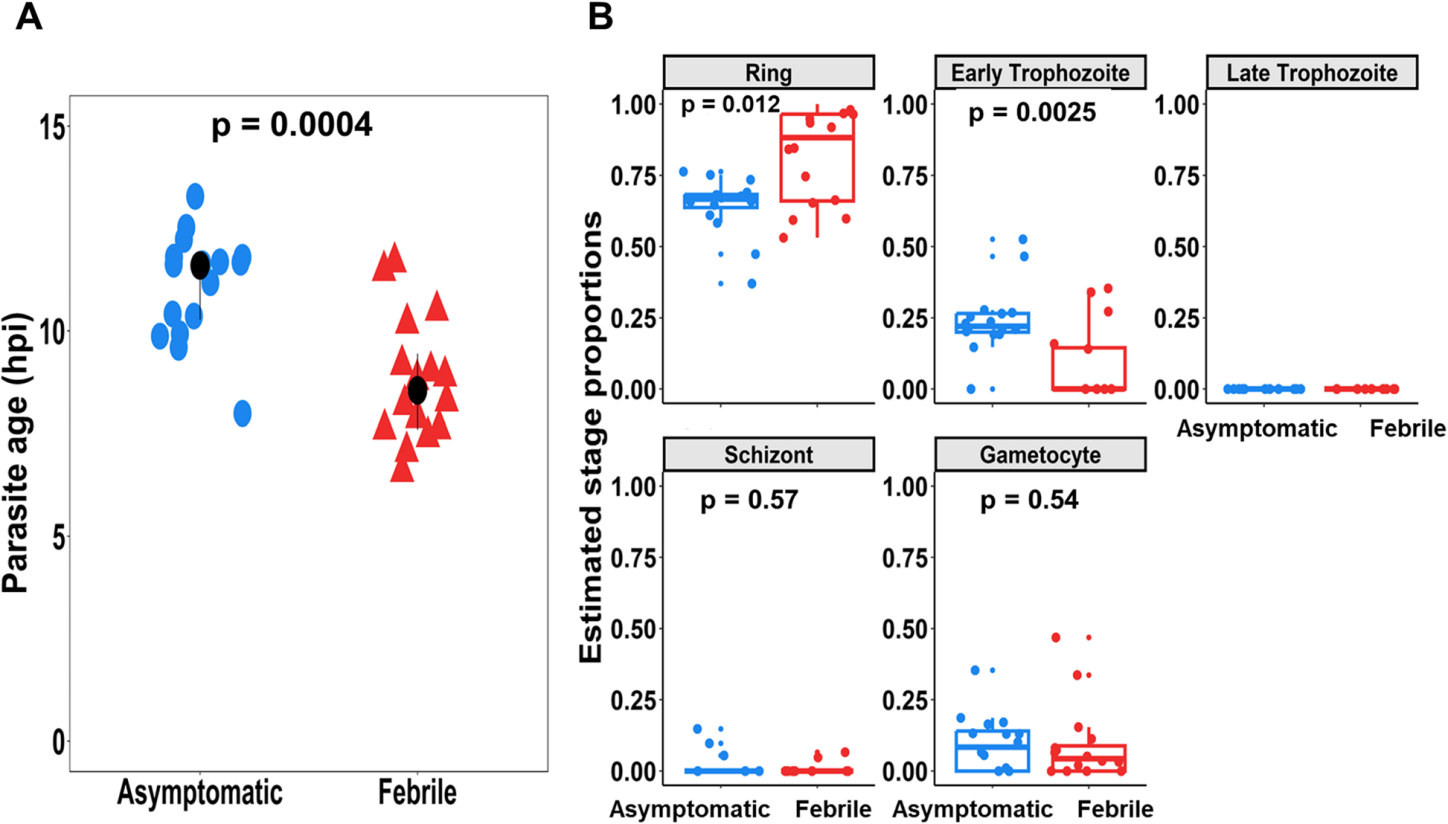
Asymptomatic infections are characterized by older, non-ring parasites compared to ensuing febrile malaria. **A** Dot plot showing the maximum likelihood estimation (MLE) of the hours post-invasion (hpi) determined using reference dataset from Bozdech et al. 2003. The samples are colored by clinical phenotype, asymptomatic infections (blue) and febrile malaria (red). The black dot shows the median hpi. **B** Boxplots showing the estimated proportions of parasite blood stage development in asymptomatic infections and febrile malaria as estimated using a mixture model and reference dataset from López-Barragán et al. (2011). For each parasite stage, Wilcoxon test (corrected for multiple testing using Benjamini & Hochberg) was performed to evaluate the difference in the proportions of parasites between asymptomatic and febrile malaria. The samples are colored by clinical phenotype, blue for asymptomatic infections and red for febrile malaria

Parasite RNA was successfully isolated from 16 asymptomatic (A) and febrile (F), AF paired samples from 12 females and 4 males. The participants had a mean age of 7.5 years (range: 0.8 – 13.4) during the asymptomatic *P. falciparum* infections and were followed for an average of 114.6 days (range: 22—235) before developing malaria symptoms. In contrast, though not significant, individuals who went from uninfected to febrile did so over an average of three months (96 days). The median parasitaemia was significantly lower during asymptomatic infections (1,720 parasites/ µl: range: 120 – 220,000) than during febrile infections (19,229 parasites/µl; range: 480 – 1,280,000) (*P* = 0.0204) (Additional file 1: Table S1). On average, approximately 15.4 million paired-end reads were generated per sample (range = 0.6 – 111 million reads). The median parasite age, as expressed in hours post-infection (hpi) and determined using the maximum likelihood estimation method [29], was statistically higher (*P* = 0.0004) in asymptomatic infections (median = 11.7 hpi, (inter-quartile range) IQR[10.48—11.9]) compared to febrile infections (median = 8.7 hpi, IQR(7.6—9.55) (Fig. 3A). The estimated proportions of each intraerythrocytic parasite stage were determined using a mixture model [31], revealing a significantly lower (*P* = 0.012) proportion of ring stage parasites in asymptomatic infections than in febrile infections (Fig. 3B). Similarly, a statistically significant (*P* = 0.0025) bias towards the early trophozoites was revealed in asymptomatic infections (Fig. 3B).

**Fig. 4:**
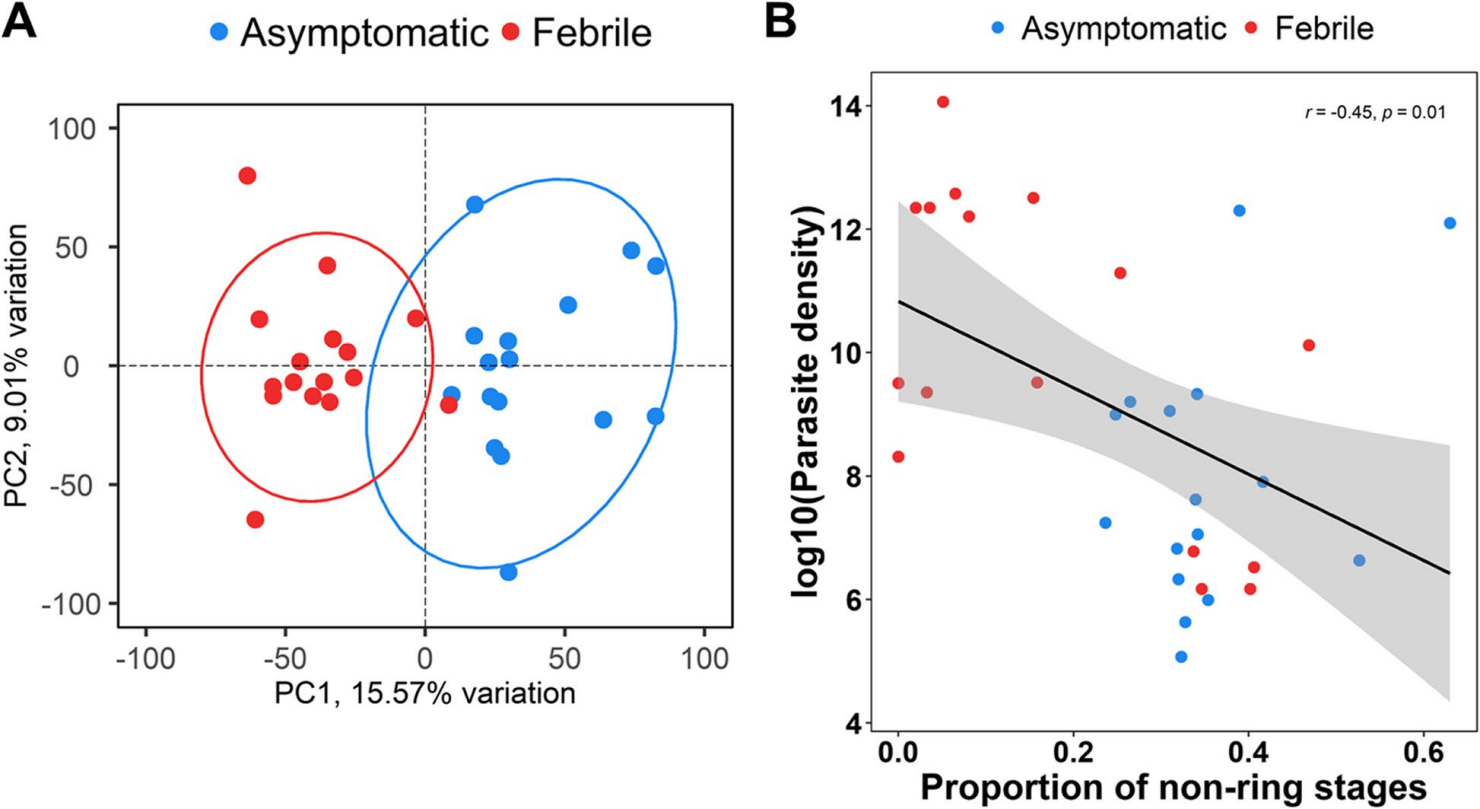
Genome-wide expression deconvolution of parasite RNAseq data. **A** Principal component analysis (PCA) of parasite RNA-seq data from paired asymptomatic and febrile malaria normalized for library size, parasite blood stage development and 3 factors of unwanted variation (normalized counts available in (Additional file 1: Table S2). The samples are colored by the clinical phenotype, blue for asymptomatic infections and red for febrile malaria. Ellipses represent 95% confidence intervals. **B** Scatter plot showing the correlation between the parasite density (log10) and the estimated proportions of non-ring stages across all the samples. The samples are colored by the clinical phenotype, blue for asymptomatic infections and red for febrile malaria

An all-gene expression principal component analysis (PCA) showed a clear distinction between febrile malaria and asymptomatic infections after correcting for library size, parasite blood stage development and unwanted variations (Fig. 4A). The proportion of the rings and non-ring stages versus parasitemia across all samples was determined whereby the proportion of non-ring parasites was significantly negatively correlated with parasitemia (r =—0.45; *P* = 0.01) (Fig. 4B). This indicates that RBCs infected with mature parasite stages circulate longer in low parasitemia.

### Host PBMC transcriptomes differentiate febrile from non-febrile malaria

A total of 49 PBMC samples from uninfected (*n* = 14), asymptomatic (*n* = 13) and febrile (*n* = 22) individuals were successfully sequenced. Of these, 20 were paired samples, i.e., 8 UF and 12 AF paired samples. The other nine samples were unpaired and comprised 6 uninfected, 1 asymptomatic and 2 febrile samples. The mean age of uninfected individuals (8.15 years) was not significantly different from that of asymptomatic individuals (7.36 years) (*P* = 0.56). There was no significant difference in parasitemia observed between the febrile infections ensuing from uninfected or asymptomatic infections (*P* = 0.504) ((Additional file 1: Table S1). On average, approximately 60 million reads mapped to each sample with a range of 11.5 – 374.4 million paired-end reads.

**Fig. 5:**
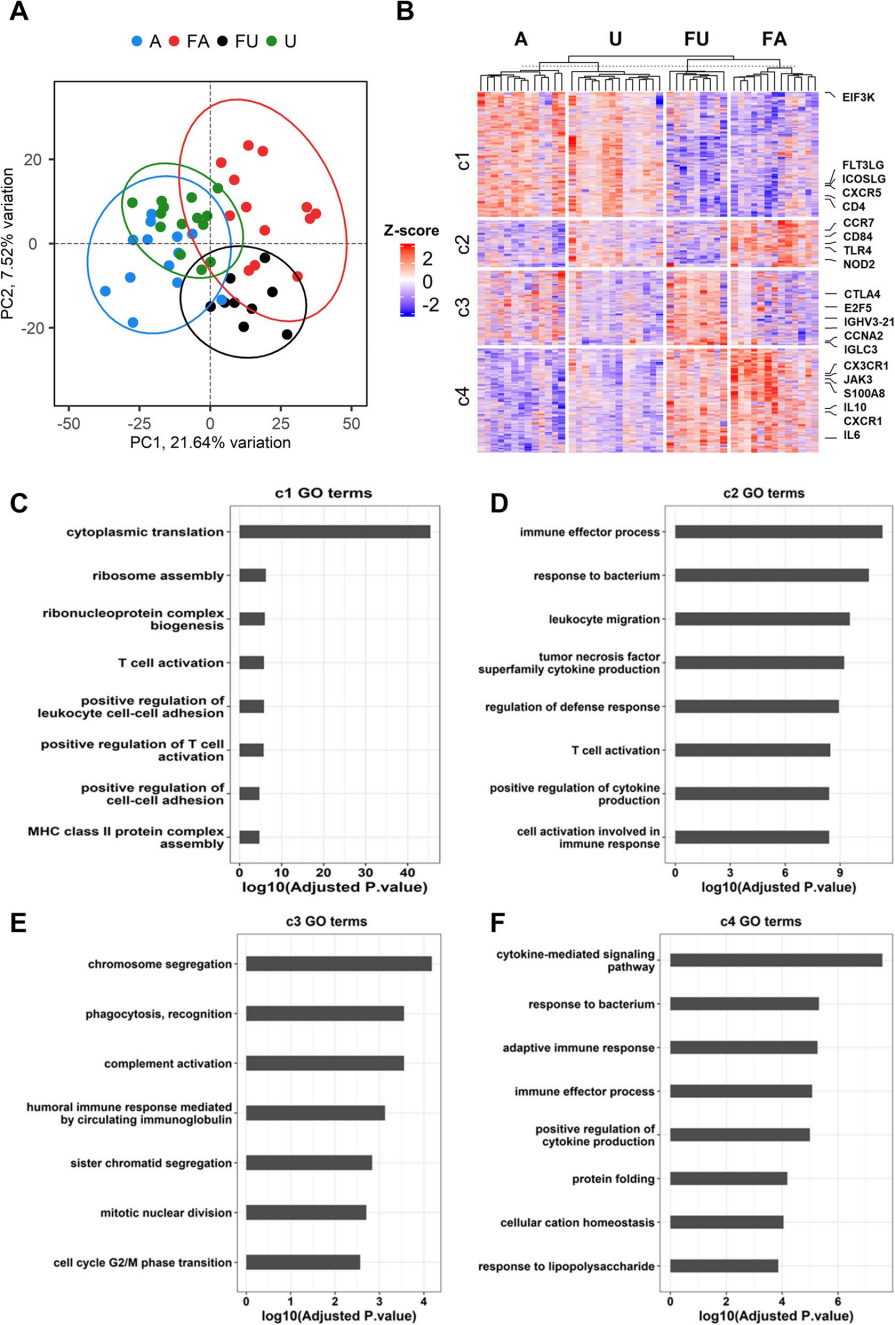
Host PBMC gene expression and functional analysis. **A** Principal-component analysis of the 4,311 differentially expressed genes (DEGs) among the four clinical phenotypes. Ellipses represent 95% confidence intervals. Percentages along x and y axes show the degree of variance explained by each principal component. The samples are coloured by the clinical phenotype i.e. A = Asymptomatic, U = Uninfected, FU = Febrile malaria ensuing from uninfected individuals and FA = febrile malaria ensuing from asymptomatic individuals. **B** Heatmap of the 4,311 DEGs (rows) among the four clinical phenotypes (columns) split into 4 clusters using K-means clustering and labelled c1 – c4. **C**, **D**, **E** and **F** Barplots showing enriched Gene Ontology (GO) terms in clusters c1, c2, c3 and c4, respectively, from the heatmap in **B**

Differential expression analysis revealed 4311 differentially expressed genes (DEGs) (Additional file 1: Table S3). Principal component analysis (PCA) using DEGs separated the samples into febrile and non-febrile groups on PC1, which was associated with 21.64% of the variation, while PC2 (7.52%) partially separated U from A and the febrile infections into those who were initially asymptomatic (FA) and uninfected (FU), suggesting a transcriptional distinction in the febrile infections (Fig. 5A). Gene clustering using DEGs identified four gene clusters 1 to 4 (c1-c4). Overall, c1 (1506 genes) was upregulated in non-febrile children (A and U groups), while c4 (1325 genes) was upregulated in febrile children (FU and FA). There was a marked upregulation of c2 (552 genes) in the FA group and a contrasting pattern of expression in c3, with an upregulation of 928 genes in FU samples (Fig. 5B).

**Fig. 6:**
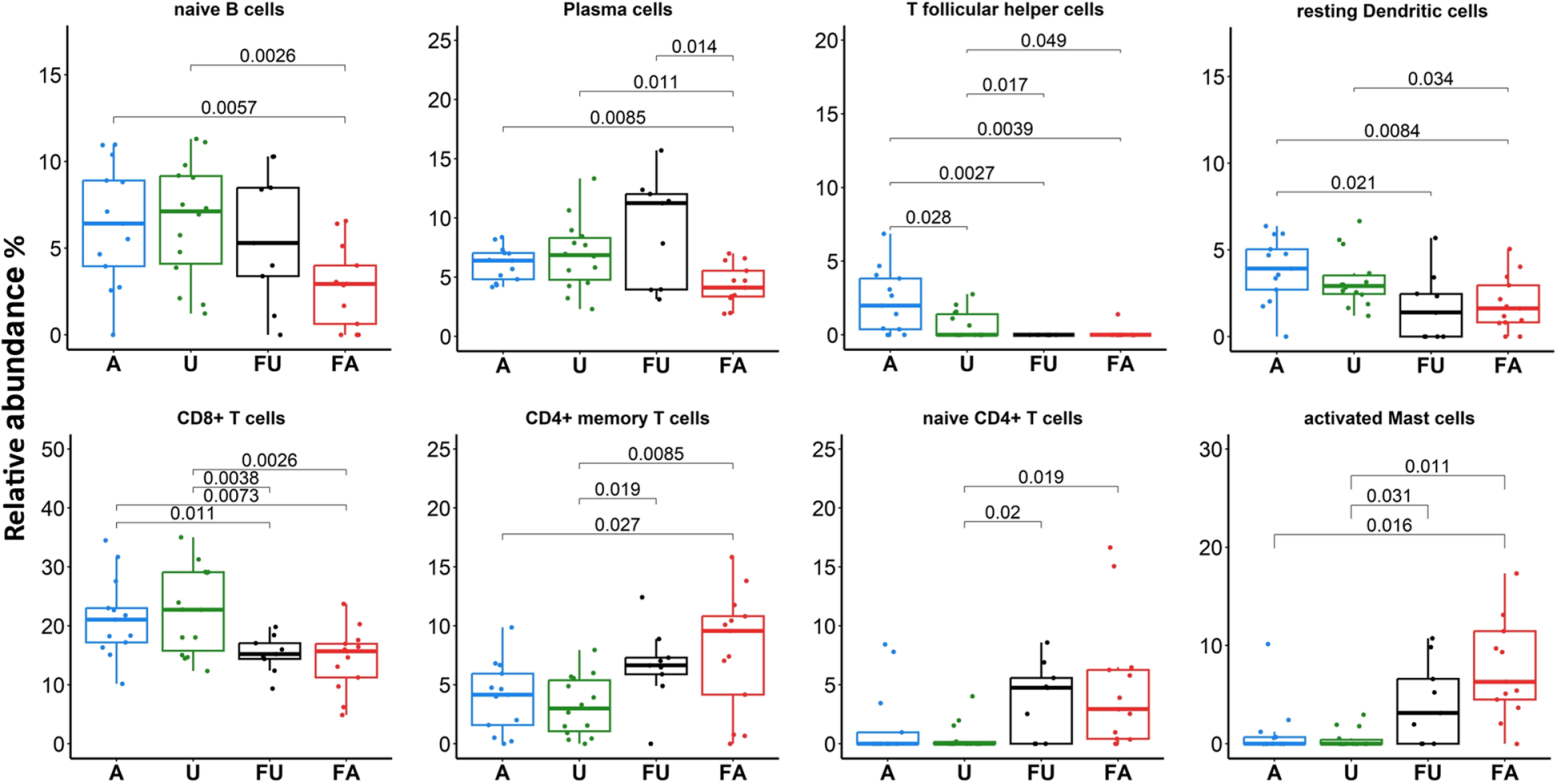
Deconvolution of PBMC cell types. PBMC subpopulation proportions were estimated for each sample using the deconvolution analysis in CIBERSORT and a gene signature matrix. Samples are coloured based on the clinical phenotype: A = Asymptomatic, U = Uninfected, FU = Febrile malaria ensuing from unifected individuals and FA = febrile malaria ensuing from asymptomatic individuals. All pairwise statistical tests indicated in the plots are Wilcoxon tests corrected for multiple testing (Benjamini & Hochberg)

Gene ontology (GO, Fig. 5C-F) and KEGG analysis (Additional file 1: Table S4) produced similar results and were used to determine the predominant functions enriched in each cluster. GO terms for cytoplasmic translation, ribosomal activity, T cell activation and cell adhesion (Fig. 5C) predominated in c1, including genes associated with protein translation factors (EIF3D, EIF3E, EIF3G and EIF3K) and ribosomal proteins (RPL18A, RPL19, RPS12, RPS16, RPL35, RPL8, RPS11, RPS27A) and genes associated with T cell activation and adhesion (CD3D, CD3E, CD3G, CD4, CD96, CD247, CD5, ZAP70, LCK, CXCR4, CXCR5, CD74 and TBX21, GATA3), Figs. 6B and C. These data suggest that protein synthesis and T cell activation were downregulated in the febrile cases.

In contrast, c4 genes were enriched for GO terms involving immune effector processes, adaptive and innate immunity and cytokine pathways (TLR2, STAT3, IL6, IL6R, ANXA3, S100A11, CXCR1, C3AR1, LRG1, S100A8, S100A12, S100A9, JAK3) and regulation of immunity (CD274, CD276, IL1RN, IL10, CR1, CD55, IDO1, IL2RA, BST2) (Fig. 5F). These data show, as expected, that markers of immune activation, particularly neutrophil are upregulated in febrile infections (FA and FU).

The deconvolution analysis revealed some significant differences in the estimated proportions of nine cell types in PBMCs among the four clinical groups. A lower proportion of naïve B cells was observed in FA, while a higher proportion of plasma cells was observed in the FU group. The proportion of CD8^+^ T cells was greatest in U and A; on the other hand, that of naïve and activated memory CD4^+^ T cells was greatest in FA and FU. A higher proportion of activated mast cells was also observed in the FA group (Fig. 6).

### Distinctions in febrile and non-febrile infections

Clusters 2 and 3 drive the separation of febrile infections based on whether they were initially from asymptomatic or uninfected individuals. Cluster 2 was enriched for GO terms involving immune effector processes, leucocyte migration, host defense and TNF production (Fig. 5D). The greatest differences in expression were in the FA group, particularly in genes associated with innate immune effector processes (TLR4, CD14, CD68 TGFB1, EOMES, CCR7, CCL3R1, CD84, TNFRSF1B, IL1B, IFNGR1). There were also some differences between the A and U groups, and a pairwise analysis of the two groups revealed an upregulation of several genes related to inflammatory responses (CCR7, CCL2, NOD2, SIRPA, TNFSF14, NFKB1) in the U group (S5 Table). The innate and inflammatory responses were strongly upregulated in FA compared to the FU group.

The GO terms enriched in c3 were nuclear division and chromosomal segregation, humoral responses, genes for pathways associated with cell cycle regulation (E2F5, CCNA2, CDC6, CDC14B, CCNB1, CDKN2C), and immunoglobulin, phagocytosis and complement (IGHM, IKC, IGHG4, IGHG2, C5) (Fig. 5E). There was a clear distinction in the gene expression of c3 genes between the FA and FU samples, where many genes were upregulated in the FU samples (Fig. 5B), indicating a relatively stronger humoral response. Pairwise analysis of the A and U groups revealed an upregulation of humoral responses (C1QB, IGHG4, IGKC, IGLC3, IGHV5-51, IGHV3-15, IGHV3-21) in the A group ((Additional file 1: Table S5).

**Fig. 7:**
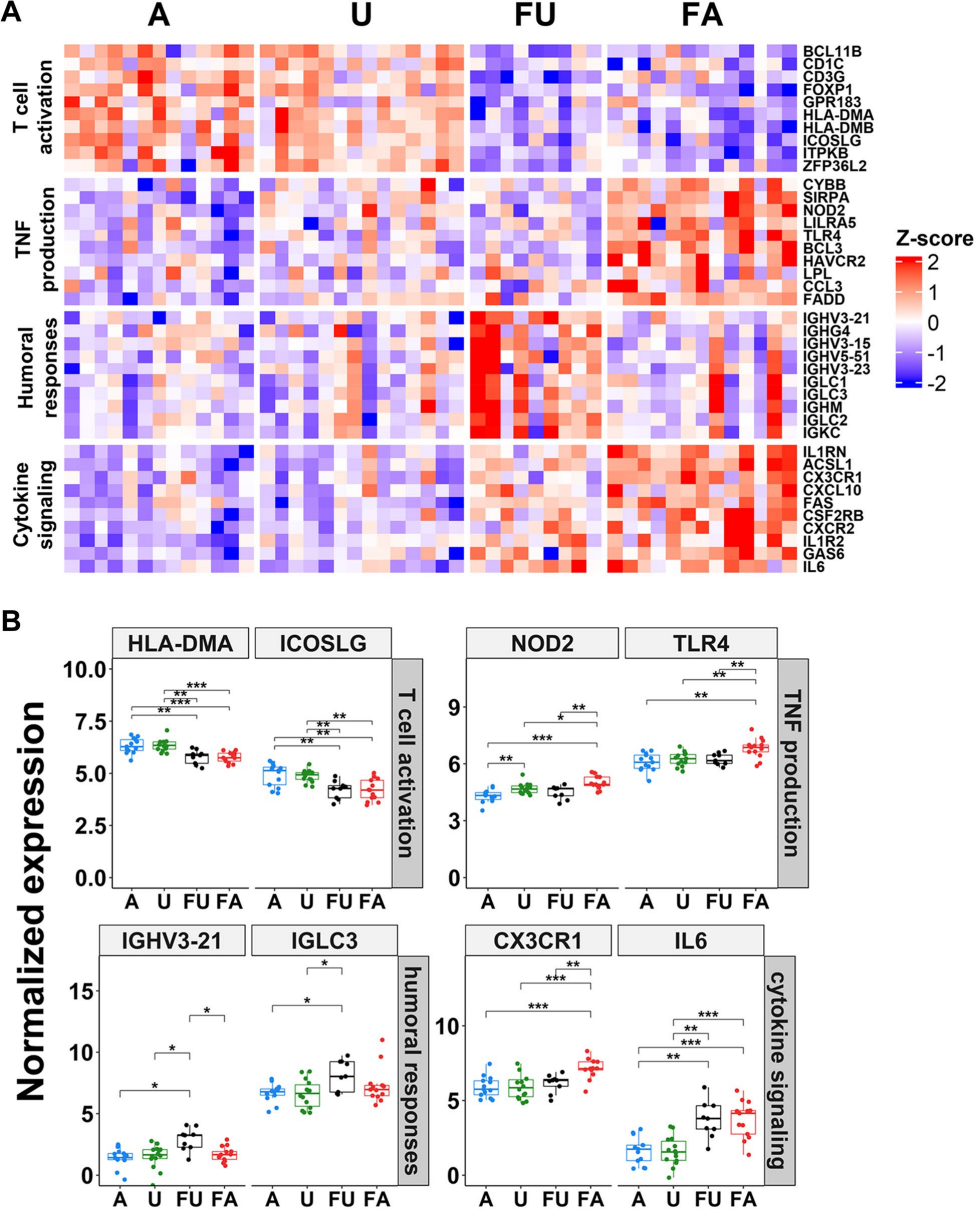
Analysis of selected Gene Ontology (GO) terms. **A** Heatmap showing the top 10 differentially expressed genes (DEGs) (based on FDR) enriched in humoral responses, cytokine signaling, TNF production and T cell activation GO terms. The genes (rows) are grouped by the clinical phenotype: A = Asymptomatic, U = Uninfected, FU = Febrile malaria ensuing from Uninfected, FA = Febrile malaria ensuing from Asymptomatic. **B** Boxplots showing the normalized expression levels of selected DEGs per GO term. The boxplots are labelled based on the clinical phenotype: A = Asymptomatic, U = Uninfected, FU = Febrile malaria ensuing from U and FA = febrile malaria ensuing from A. Pairwise statistical analyses indicated in the plots are Wilcoxon tests corrected for multiple testing (Benjamini & Hochberg, * =  < 0.05, ** =  < 0.01 and *** =  < 0.001)

A summary of the 10 most highly differentially expressed genes for the gene ontology terms, humoral responses, cytokines, TNF and T-cell activation in each group is shown in Fig. 7A, with a comparison of the normalized expression of a selection of relevant genes (Fig. 7B). Both the A and U samples show upregulated T-cell activation compared to the respective febrile samples. The FU samples had the strongest expression of immunoglobulin-related genes and humoral response whereas the FA samples had the greatest gene expression for cytokines and TNF production.

### PBMC proteome distinguishes uninfected, asymptomatic and febrile states

**Fig. 8:**
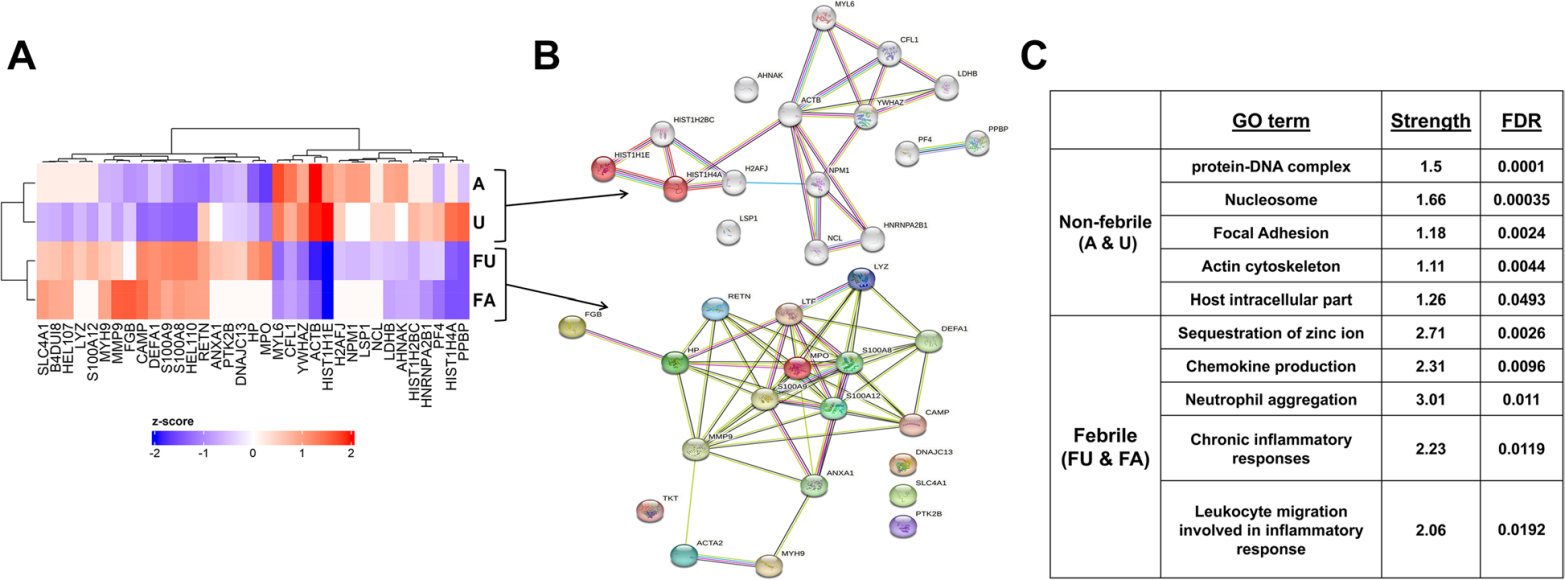
Differential expression of proteins among FA and FU pairs. **A** Heat map of the 35 significantly differentially expressed protein in the clinical phenotypes identified using isobaric labeling-based on quantitative proteomics. The columns represent the proteins and the rows the clinical phenotypes, A (Asymptomatic), U (Uninfected) and F (Febrile from Asymptomatic [FA] and Uninfected [FU]). **B** Protein–protein interaction network analysis using STRING database. Proteins are shown as nodes, and the color of each link is based on the type of evidence available for the interaction between two proteins (e.g., purple- experimental, light blue – homology, blue – coocccurence, black – coexpression, purple – experimental, green – text mining). **C** Gene ontology analysis in STRING database. Strength is a measure of the enrichment effect while FDR is the adjusted *P*-value after correcting for multiple testing using the Benjamini-Hochberg

A paired analysis of PBMC samples from 16 FU pairs and 22 FA pairs was performed using TMT proteomics technology to examine differentially expressed proteins. A total of 318 proteins were identified (q-value < 0.1) after filtering out contaminant proteins. Differential protein expression analyses identified 35 proteins (Fig. 8A and Additional file 1: Table S6). Protein–protein interactions (PPI) analysis in the STRING database revealed significant enrichment of proteins in either the febrile or non-febrile groups (PPI enrichment *P* value < 0.001) (Fig. 8B). Consequently, 16 proteins were upregulated in the non-febrile samples and were enriched for protein-DNA complex and nucleosome (HIST1H1E, HIST1H2BC, H2AFJ, HIST1H4A) cellular components (Fig. 8C). The remainder were upregulated in febrile infections and were enriched for chemokine production (MPO, HP, S100A8, MMP9, ANXA1, DEFA1), neutrophil aggregation and chemotaxis (S100A8, S100A9, S100A12) and chronic inflammatory response (CAMP, S100A8) biological processes (Fig. 8C). Although a low number of proteins were detected, it indicates chromatin changes in asymptomatic and uninfected individuals, and innate immune responses in febrile infections.

## Discussion

The longitudinal cohort provided a unique opportunity to follow up individuals with asymptomatic *P. falciparum* infections until they developed symptomatic malaria. The parasite transcriptomic profile in these individuals highlighted an association between non-ring stages and lower parasitemia levels. This is consistent with the abundance of trophozoite stages (average of 12 hpi) in asymptomatic infections. In contrast, febrile infections showed a bias towards ring stage parasites. These observations have been made before in a similar study in Mali [5], reinforcing the idea that parasites adapt to their host, irrespective of differences in malaria transmission. Malaria transmission in Mali is defined by a sharply distinct dry season with very little malaria transmission that switches in the rainy season to a massive upsurge in malaria infections [47]. Along, the Kenyan Coast, the study region, malaria transmission is essentially perennial, demonstrating that the malaria parasite adapts by modifying its host to ensure sustained malaria transmission and parasite survival. This is further demonstrated by a similar profile of increased ring stage parasites previously observed in severe and cerebral malaria cases compared to those from non-severe malaria [31, 48]. The latter infections were skewed towards the early trophozoite stages [31]. The survival of the parasite that is linked to the different portrayal of erythrocyte parasite stages and the disease spectrum (asymptomatic, mild and severe) has previously been attributed to parasite virulence and the ability of the parasite to cytoadhere to host epithelial cells, which potentially dictates the severity of malaria infection outcomes [49]. Increased cytoadherence may result in higher parasitemia and increased malaria severity [5]. This brings to the fore the potential role of VSAs; antibodies against these proteins have been shown to select against parasites with high binding ability [50, 51], hence inadvertently selecting for parasites with low binding ability in semi-immune asymptomatic carriers. These parasites with lower binding ability will predominate and circulate for a longer time in the bloodstream, increasing the splenic clearance of infected erythrocytes [5] and reducing parasitemia. The potential role of VSAs in maintaining asymptomatic infections should be examined further, since it was not within the scope of this study. An important contributing factor to the differences between asymptomatic and febrile infections could be how the parasite adapts to the host environment which is still not well understood [52–54].

The febrile infections featured a significant increase in parasitemia, which coincides with a preponderance of ring stage parasites, agreeing with similar studies in Mali [5] and Cameroon [55]. The high levels of parasitemia and superinfection with new clones (recently demonstrated in this cohort using *msp2* genotyping and amplicon sequencing of *ama1* [15, 16]) is likely to expose the host to parasite components unfamiliar to the immune system, stimulating more of a ‘primary’ inflammatory-type response, facilitating some control of the high parasitemia clearance, but which may contribute to symptomatic/febrile malaria [56]. The analysis of gene expression profiles from PBMCs in this study, showed that febrile infections were indeed characterized by the upregulation of immune pathways related to immune effector functions, production of inflammatory cytokines, and humoral responses. These results agree with previous studies that compared pre-infection and early febrile malaria infection and revealed a marked activation of pro-inflammatory response pathways in febrile infections [22, 23, 57].

The transcriptomic analyses of febrile infections were surprisingly segregated based on whether the febrile malaria infection was preceded by an asymptomatic infection or from those who were uninfected. Febrile infections ensuing from asymptomatic infections were characterized by a greater enrichment of genes involved in innate immune processes. In contrast, febrile infections in previously uninfected individuals were characterized by a greater upregulation of humoral immune responses. The increase in transcripts associated with humoral immunity could be due to the higher proportions of plasma cells seen in the deconvolution analysis rather than increased transcription on a per-cell basis; single-cell gene transcriptional analysis would clarify this. Strong inflammatory responses have previously been shown to impair the ability of T follicular helper cells to activate B cells to produce antibodies in the germinal centers [58–60]. Altogether, it is not clear why febrile infections in previously uninfected individuals showed a greater upregulation of gene expression characteristic of B cells and humoral responses. However, plasma antibody responses were not measured, which might reflect more accurately the humoral response. It could be, as suggested by the RNA data, that IgM producing B-cells characteristic of a more primary response, are elevated in the uninfected group, suggesting a less well-developed state of acquired immunity. A longitudinal study incorporating parasite levels, cellular studies and antibody responses would go some way to addressing this.

Prior to febrile infections, the gene expression profiles of asymptomatically infected children and uninfected children were broadly similar. However, the asymptomatic samples did show greater expression of some genes associated with the humoral response compared to uninfected children, perhaps indicating that persistent infection may stimulate greater antibody responses [61]. It is also possible that the immune response has been modified to tolerate the continued parasite presence, and therefore, this would be important to investigate in detail. Our data are similar to previous observations showing gene pathways involved in the production of pro-inflammatory cytokines that were least activated in asymptomatic individuals compared to uninfected individuals [22] and febrile malaria [21].

## Conclusions

Asymptomatic carriers are associated with downregulation of inflammatory responses. In contrast, their ensuing febrile infection appeared to elicit increased inflammatory responses compared to febrile infections ensuing from uninfected individuals. The clear segregation of febrile outcomes based on whether the preceding state was uninfected or asymptomatic provides further evidence that the presence of parasitemia in asymptomatic individuals modifies the host, offering an immunological advantage to the children. However, impaired antibody responses among asymptomatic individuals upon superinfection align with the reduced malaria vaccine efficacy observed among malaria exposed individuals icon endemic regions. Therefore, to achieve malaria elimination, malaria control campaigns should aim to eliminate all parasites, including those in asymptomatic individuals.

### Supplementary Information

**Additional file 1: S1 Table. **Participants metadata. **S2 Table.** Normalized parasite RNAseq read counts of 16 AF pairs. **S3 Table.** Differentially expressed host PBMC genes across A, U and F groups. **S4 Table.** Functional GO and KEGG analysis of host PBMC genes. a) KEGG analysis results from genes differentially expressed in each cluster. b) Gene Ontology (GO) analysis results from genes differentially expressed in each cluster. P.adjusted represents the Benjamini-Hochberg adjusted P-values and was used to sort the GO terms. BP represents the Biological Process category. **S5 Table.** Differentially expression analysis of host PBMC genes between A and U groups. **S6 Table.** Differentially expressed analysis of host PBMC proteins across paired AF and UF groups.

## Data Availability

Parasite and host RNAseq data and processed counts have been deposited in the National Center of Biotechnology Information’s Gene Expression Omnibus (GEO) available under GEO accession number GSE240643 and GSE241467, respectively. All mass spectrometry raw files associated with this study are provided in the online Dryad repository (https://datadryad.org/stash/share/8jLf_ceeQfJ8B1FTcGlYzXYu-RQ4c7yddZw2PA4BugE).

## Abbreviations

PBMCs: Peripheral blood mononuclear cells
VSAs: Variant surface antigens (VSAs)
Hpi: Hours post-infection
DEGs: Differentially expressed genes
IQR: Interquartile range
TMM: Trimmed mean of M values
PCA: Principal component analysis
PPI: Protein–protein interactions
GO: Gene Ontology
KEGG: Kyoto Encyclopedia of Genes and Genomes
